# Cross species transmission of pseudorabies virus leads to human encephalitis and visual impairment: A case report

**DOI:** 10.3389/fneur.2022.950931

**Published:** 2022-09-20

**Authors:** Hui Huang, Na Wang, Zhi-Bing Ai, Jun Chen, Wei Huang, Yi Bao

**Affiliations:** Department of Neurology, Taihe Hospital of Shiyan, Affiliated Hospital of Hubei Medical University, Shiyan, China

**Keywords:** pseudorabies virus, viral encephalitis, secondary epilepsy, NGS, endophthalmitis, cerebrospinal fluid

## Abstract

Pseudorabies virus (PRV) is a common pig infectious disease. There have been few reports of PRV infection in humans. The patient in this article had acute onset, which was manifested by fever, epilepsy, disturbance of consciousness, and other symptoms. The disease progressed rapidly and worsened in a short time so the ventilator had to be used to assist breathing. In the later stage of treatment, serious visual impairment also occurred. Pseudorabies virus was found in cerebrospinal fluid by second-generation gene sequencing (NGS). This indicates that the pseudorabies virus can spread across species, leading to human encephalitis and severe visual impairment. Therefore, attention should be paid to this disease, active prevention, and early detection are helpful to improve the treatment effect.

## Introduction

Pseudorabies virus (PRV) is also known as porcine herpes virus type I, infectious bulbar paralysis virus, and so on. PRV is very common in pig infection, but there are few reports on PRV-infected people (1, 2). We evaluated a case of encephalitis with unknown etiology, rapid onset, severe clinical manifestations such as fever, epilepsy, disturbance of consciousness and visual impairment, and detected pseudorabies virus by second-generation sequencing (NGS). The report is as follows to improve clinicians' understanding of the disease.

## Case report

On the evening of 14 April 2021, a 54-year-old male did not know that his slipper had fallen while walking, and did not respond to the family's inquiries. At about eight o 'clock in the evening, the patient had a fever, with the highest temperature of 41°C. After the local hospital gave diclofenac sodium symptomatic antipyretic, the temperature gradually decreased to normal. During this period, the patient did not answer questions.

At about 10 o'clock in the evening, the patient began to lift the right upper limb involuntarily. The next morning, the patient was found to have a poor response. He could open his eyes when people called him, but did not respond, accompanied by involuntary twitching of the right limb. There was no nausea, vomiting, palpitation, shortness of breath, and incontinence during the course of the disease. To seek further diagnosis and treatment, he came to our hospital and was admitted to our department by the outpatient clinic with “consciousness disorder to be examined.” Past history: found diabetes for half a month, denied any other special history. Physical examination: T 37.3°C, P 98 times/min, R 20 times/min, BP 129/95 mmhg, drowsiness, no obvious abnormalities found in skin, mucosa, lymph nodes and cardiopulmonary abdominal examination, equal size and equal circle of bilateral pupils with 3 mm in diameter, slow light reflex, involuntary twitching of right limb, negative meningeal irritation sign, and no positive signs found in other nervous systems physical examination. Initial diagnosis of consciousness disorder to be examined: intracranial infection, secondary epilepsy?

**Figure 1:**
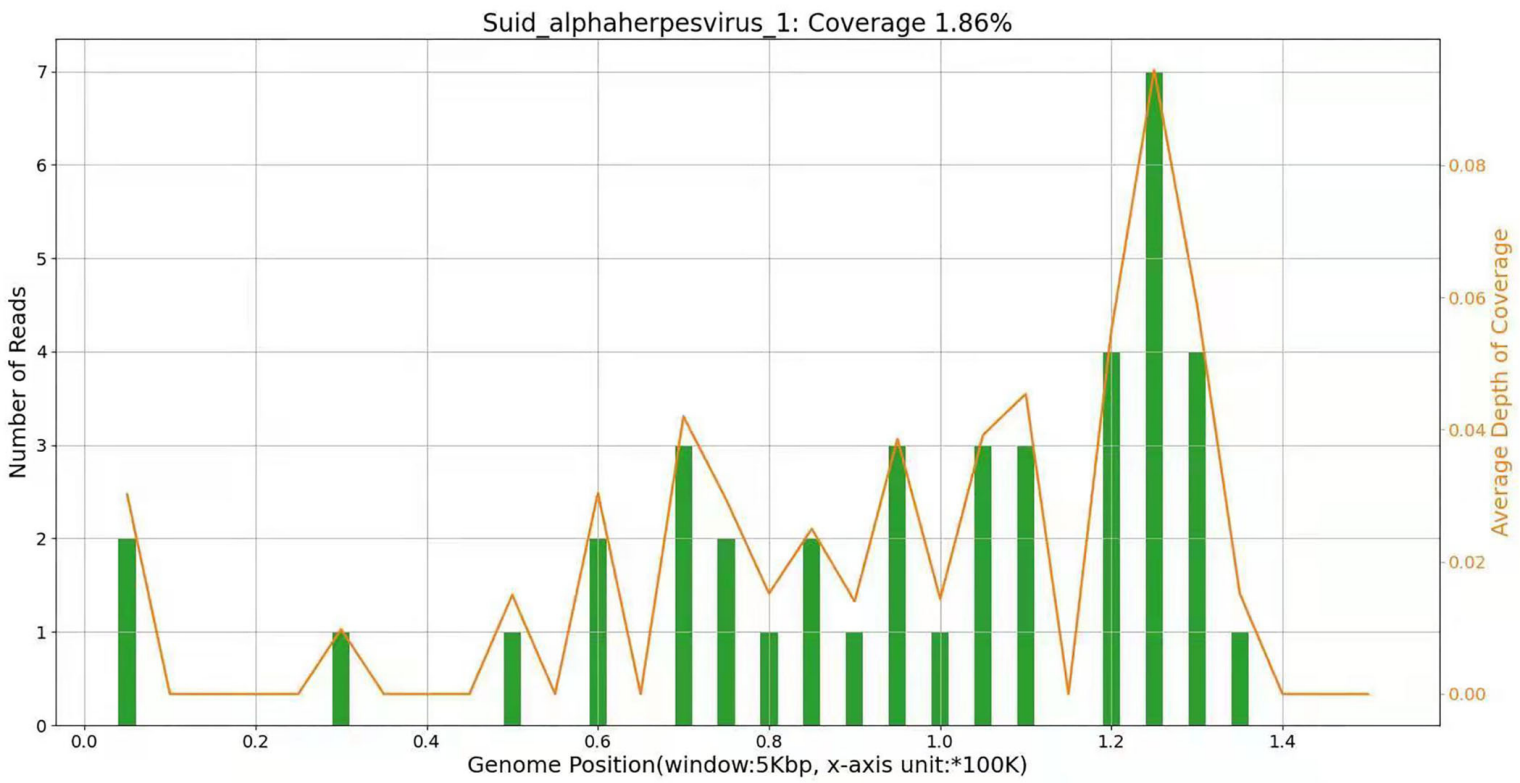
On 15 April, NGS results of cerebrospinal fluid showed that the pathogen of encephalitis in this patient was pseudorabies virus, and the genome coverage rate was 1.86%.

Improve related examinations, blood routine: WBC 8.7 g/L, NE 76.4%, HGB 163 g/L, PLT 111 G/L, blood biochemistry: TBil 53.4 umol/L, NCBil 43.3 umol/L, SAA 21.4 mg/L. There were no abnormalities in the electrolyte, liver function, renal function, blood glucose, high-sensitivity C-reactive protein, erythrocyte sedimentation rate and coagulation function, as shown in Table 1. Electroencephalogram (EEG): the main rhythm of each lead was 10 HZα with medium and low amplitude, and the parietal and occipital region was dominant, with left and right asymmetry and poor amplitude modulation. The left lead had more rhythm and activity of 4–7 HZθ with medium and low amplitude, and slightly more activity of 3–3.5 HZδ, and the front head was very biased. Brain magnetic resonance imaging (MRI): multiple spots and bands of abnormal signals were found in the left insula and temporal cortex, T2WI and T2 flair showed hyperintensity, T1 flair showed isointensity and hypointensity, and GD-DTPA enhanced scanning showed no obvious abnormal enhancement, as shown in Figure 1. Cranial MRA, MRV, neck vascular color ultrasound, chest CT, electrocardiogram, and heart color ultrasound showed no abnormalities. The patients were treated with epilepsy control, brain edema prevention, brain cell nutrition, anti-infection, water and electrolyte balance and liver protection, and closely monitor the changes of vital signs and mental pupils.

**Table 1:** Laboratory dynamic examination results of patients with pseudorabies virus encephalitis.

**Test items**	**Apr 14**	**Apr 16**	**Apr 17**	**Apr 19**	**Apr 23**	**Apr 28**	**May 12**	**Jun 4**
WBC (G/L)	8.7	13.1	8.9	5.6	8.7	9.4	4.5	4.7
NEU (%)	76.4	91.5	93.1	91.6	85.4	82.2	59.8	69.9
HGB (g/L)	163	152	146	148	137	120	117	111
PLT (G/L)	111	115	108	108	76	126	132	128
K^+^ (mmol/L)	4.2	3.9	3.8	3.9	3.9	4.1	3.3	3.7
Na^+^ (mmol/L)	136.5	140.6	147.4	152.4	144.1	139.1	138.1	139.4
ALT (U/L)	14.3	14.4	14.1	19.6	175.7	28.4	7.9	15.9
AST (U/L)	21.5	-	13.7	27.1	137.1	46.6	21.4	17.4
γ-GT (U/L)	25.9	19.9	22.4	29.7	76	51.1	50.1	39.1
ALP (U/L)	52.9	47.8	54.7	58.8	64.9	58.5	47.8	48.2
Alb (g/L)	40.7	36.2	33.7	31.3	31.1	38.2	37.1	38.9
A/G	1.3	1.4	0.8	0.6	0.6	1.1	1.3	1.6
Glu (mmol/L)	9.5	11.3	14.7	20.5	11.6	7.4	3.3	6.5
TBil (umol/L)	53.4	12.1	15.1	10.6	14.9	16.4	12.4	16.4
NCBil (umol/L)	43.3	0.8	11.4	7.2	9.8	11.7	10.3	13.3
CBil (umol/L)	10.1	11.3	3.7	3.4	5.1	4.7	2.1	3.1
Urea (mmol/L)	5.9	7.1	9.5	14.5	10.7	4.9	3.9	2.6
Cr (umol/L)	82.1	86.8	82.9	89.1	75.7	64.7	80.3	48.9
UA (umol/L)	328.7	158.7	262.6	411.8	317.5	214.1	268.3	258.2
NH3 (umol/L)	18.3	18.2	7.1	33.3	23.9	11.7	13.4	18.3
PA (mg/L)	436.2	78.3	149.2	195.1	215.6	222.8	195.3	204.1
Osm (mmol/L)	297	307	327	348	318	299	290	295
hsCRP (mg/L)	3.1	82.9	53.7	-	-	-	5.9	-
5-NT (U/L)	1.8	1.6	1.3	0.4	0.5	1.7	2.1	2.1

On 15 April, the patient was drowsy, still had intermittent twitching of the right lower limb, and coffee residue-like substances were extracted from the gastric tube. It was considered that stress ulcers occurred, and stomach protection drugs were added. In order to find out the cause, a lumbar puncture was performed to check the cerebrospinal fluid, and the pressure was 300 mm water column. The routine and biochemical results of cerebrospinal fluid are shown in Table 2. The diagnosis was considered viral encephalitis or autoimmune encephalitis, and antiviral combined with gamma globulin were added to the treatment. The serum and cerebrospinal fluid samples were sent to BGI for etiological second-generation sequencing examination, and the cerebrospinal fluid and serum were sent to kangshengda company for autoimmune encephalitis antibody detection. Dynamic monitoring of blood routine, electrolyte, liver and kidney function, blood glucose, CRP, and other related indicators during hospitalization are shown in Table 1. On 16 April, the patient was delirious, with a Glasgow (GCS) score of 3 points. He still had intermittent right limb twitching, intermittent fever, thick breathing sound, decreased blood oxygen saturation, and it was difficult to maintain stable vital signs. So he was urgently transferred to the central intensive care unit (ICU), and ventilator are assisted breathing was performed after endotracheal intubation.

**Table 2:** Cerebrospinal fluid examination results of patients with pseudorabies virus encephalitis.

**CSF test items**	**Apr 15**	**Apr 22**	**May 4**	**Jun 4**
Pressure(mmH_2_O)	300	220	140	130
Color	No	No	No	No
Transparent	Yes	Yes	Yes	Yes
Clot	No	No	No	No
WBC (10^∧^6/L)	39	37	31	27
Monocytes (%)	89	96	98	
Multilobar nuclear cells (%)	11	4	2	
PRO (g/L)	0.23	0.29	0.45	0.39
Cl (mmol/L)	126.68	136.48	126.08	131.38
Glu (mmol/L)	6.21	5.41	2.75	3.89
LDH (U/L)	14.8	29.6	13.8	15.7
ADA (U/L)	1.5	1.5	0.3	4.7

**Figure 2:**
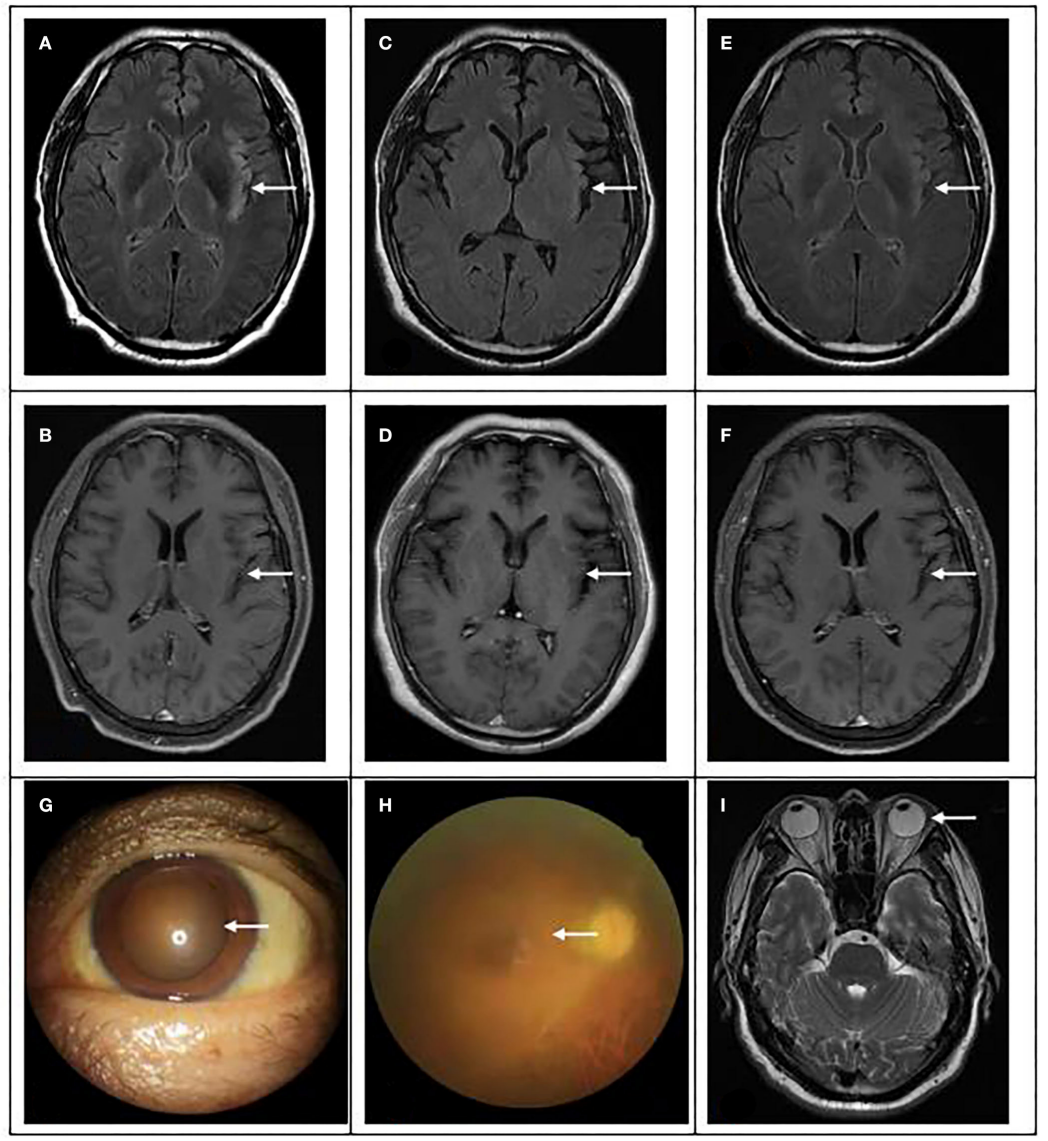
Brain magnetic resonance and eye examination results. **(A,B)** On 15 April, brain magnetic resonance showed multiple spots and bands of abnormal signals in the left insular and temporal cortex, high signal in T2WI and T2flair, and equal and low signal in T1flair. **(C,D)** On 24 April, MRI showed abnormal signal and meningeal changes in the left frontal temporal insula. **(E,F)** On 04 June, magnetic resonance imaging showed that the range of inflammatory lesions was slightly smaller than before. **(G)** The pupil of the patient was significantly enlarged, with a diameter of 5 × 5 mm. **(H)** Fundus lesions: vitreous cavity is turbid, the optic disc boundary is indistinctly seen, and white line changes can be seen in the peripheral momentum. **(I)**: No abnormality was found in eyeball magnetic resonance.

On 19 April, the patient became drowsy. After people shouted, he could open his eyes and move according to the instructions. He still had an intermittent fever, the highest temperature was 38.6°C, there was no headache, the activity of the left limb was normal, the muscle strength of the right limb was grade 3, and there was no limb convulsion. Spontaneous breathing had been restored and the ventilator had been evacuated, but it is still in the state of endotracheal intubation. The second generation sequencing results of cerebrospinal fluid showed that it was infected with pseudorabies virus with a coverage rate of 1.86%, see Figure 2 for details. There was no abnormality in the second-generation sequencing of serum, and the antibodies of cerebrospinal fluid and serum to autoimmune encephalitis were negative. After inquiring about the medical history, we learned that the patient had been engaged in the pig industry for a long time. The patient had a clear diagnosis of viral encephalitis and continues to receive antiviral treatment. Considering that some of the patients were prone to retinitis leading to blindness, we invited ophthalmology to consult. Eye ophthalmologist examination: there was no congestion in the conjunctiva, the cornea was transparent, and the pupils of both eyes were very small, about 1.5 × 1 5 mm, direct and indirect light reflection existed, and it was difficult to peep into the fundus through ophthalmoscopy. On 22 April, the lumbar puncture was rechecked, the pressure was 220 mm water column, the routine and biochemical results of cerebrospinal fluid were not significantly changed. On 24 April, the patient's mind improved, the activity of the right limb was still poor, and the pupils on both sides were equal round, with a diameter of 3 mm. Re-examination of cranial MRI showed that the left frontotemporal insula had abnormal signals and meningeal changes, and the possibility of inflammatory lesions was high, which was better than before. On 27 April, the patient still had fever, the highest body temperature was 37.8°C, and the right muscle strength was grade 3. After pulling out the endotracheal intubation, the respiration was steady.

On 29 April, the patient could speak clearly, but the speech did not accord with the scene, and he was restless intermittently. Physical examination showed that memory and calculation ability were decreased, bilateral pupils were equal in size and circle, 5 mm in diameter, right muscle strength was grade 4, Risperidone was added for symptomatic treatment, and the rest remained unchanged. Therefore, we asked for ophthalmic consultation. Ophthalmic examination: VOD: 0.25, VOS: finger/2.5 m, fixed pupils in both eyes, mydriasis, 5 × 5 mm, fundoscopy showed that the retina at the posterior pole was flattened, no obvious hemorrhage or exudation was found. Therapeutically, antiviral eye drops and other local treatments were administered locally.

On 04 May, the patient was conscious, without irritability, and the muscle strength of the right limb was further improved, but the vision was still unclear. After reexamination of lumbar puncture, the cerebrospinal fluid pressure was 140 mm water column, the protein content increased, and there was no significant change in the rest. On 5 May, invited ophthalmology to consult again. Ophthalmic examination: VOD: 0.1 (+ 4.75d / + 1.25d × 155 corrections no response), VOS: 0.1 (+ 5.00d / + 1.50D × 180 corrections no response); Intraocular pressure: od 14 mmHg, os 14 mmHg; There was no congestion in the conjunctiva of both eyes, the cornea was transparent, the anterior chamber was deep, the crystal was transparent, the pupils of both eyes were fixed and dilated, 5 × 5 mm, the pupil margin was adhered to the front surface of the lens, pigmented keratic precipitates (KP) could be seen in the pupil area, fundus: vitreous cavity was turbid, the boundary of the optic disc could be seen faintly, and white linear changes could be seen in the peripheral omentum; The right eye macular OCT showed that the morphology of the right eye fovea was recognizable, the inner surface of the macular retina was still smooth, and the local ellipsoid structure was slightly disordered, see Figure 2 for details. The OCT of the left eye macular did not cooperate. B-ultrasound showed vitreous opacity in both eyes. It is necessary to continue to give eye drops to the patient for treatment.

On 12 May, the patient complained of transient dizziness when he got up and moving, which disappeared after standing. Both eyes were still blurred, bilateral pupils were dilated to the edge, the light reflex disappeared. The patient asked to be transferred to a superior hospital for treatment. On 3 June, the patient came to the hospital for follow-up visit, and the pathological changes in both eyes were no better than before. Reexamination of MRI showed that the range of inflammatory lesions was slightly smaller than before.

## Discussion

Pseudorabies virus belongs to a herpesvirus subfamily in Herpesviridae, which is round or oval in shape. It is composed of double-stranded DNA, 20 hedral nucleocapsid, envelope, and lipid bilayer from inside to outside. The size of double-stranded DNA is about 150 kb and the content of G + C is 74%; The envelope is located in the outermost layer of virus particles and consists of 11 glycoproteins and 4 non-glycoproteins. It plays an important role in the process of PRV infecting host cells. PRV is widely distributed all over the world, and pigs are the main natural host and source of infection. PRV is neurophilic and can retrogradely infect the nervous system from postsynaptic to presynaptic neurons, which can cause pseudorabies (PR). The clinical symptoms of sick pigs are very serious. The main pathological changes are encephalomyelitis, ganglionitis, hemorrhagic pneumonia, and necrotizing lymphadenitis, which can lead to death in severe cases (1, 2).

In recent years, studies have found that PRV can cause cross-species transmission and induce human infection. In Mravak et al. (3), reported three suspected PRV infection cases with positive serum antibodies. All three patients showed prodromal symptoms after close contact with livestock for 1–3 weeks, followed by self-limited multi-cranial nerve involvement, suggesting that PRV can infect humans (3). In 2018, a pig farmer developed fever, headache, and visual impairment. AI JW et al. detected the unique PRV gene sequence using NGS, and detected the presence of PRV DNA in the patient's vitreous humor using real-time PCR. PRV antibody was detected from the plasma 4 months after the patient's onset, confirming that PRV can spread and infect humans across species (4). In 2018, all four patients developed a high fever, headache and chills after occupational exposure to raw pork, and epilepsy, coma and respiratory failure occurred rapidly within 1–4 days. CSF analysis showed a slight increase in leukocyte count, and cranial MRI showed high T2 weighted values of bilateral temporal lobes and basal ganglia. PRV sequences were detected in cerebrospinal fluid samples of two patients through NGS. Therefore, Zhao et al. reported for the first time that PRV can cause human encephalitis (5). In 2019, Yang HN et al. reported a case of human PRV encephalitis in China. A 43 year old patient developed headache, convulsion, and coma within 3 days after contacting raw pork. Plain CT scan of the brain showed low density in the left marginal lobe, bilateral basal ganglia and occipital lobe. PRV nucleic acid sequence and PRV antibody were detected in the patient's cerebrospinal fluid, confirming PRV encephalitis (6). In addition, there were also cases reported that patients had acute fever, disturbance of consciousness, convulsions and respiratory failure, and some cases had retinitis, which worsens rapidly. Severe cases needed to be admitted to ICU to use mechanical ventilation to assist breathing (7–9). In conclusion, the cross species transmission of PRV to humans is gradually confirmed by research.

**Figure 3:**
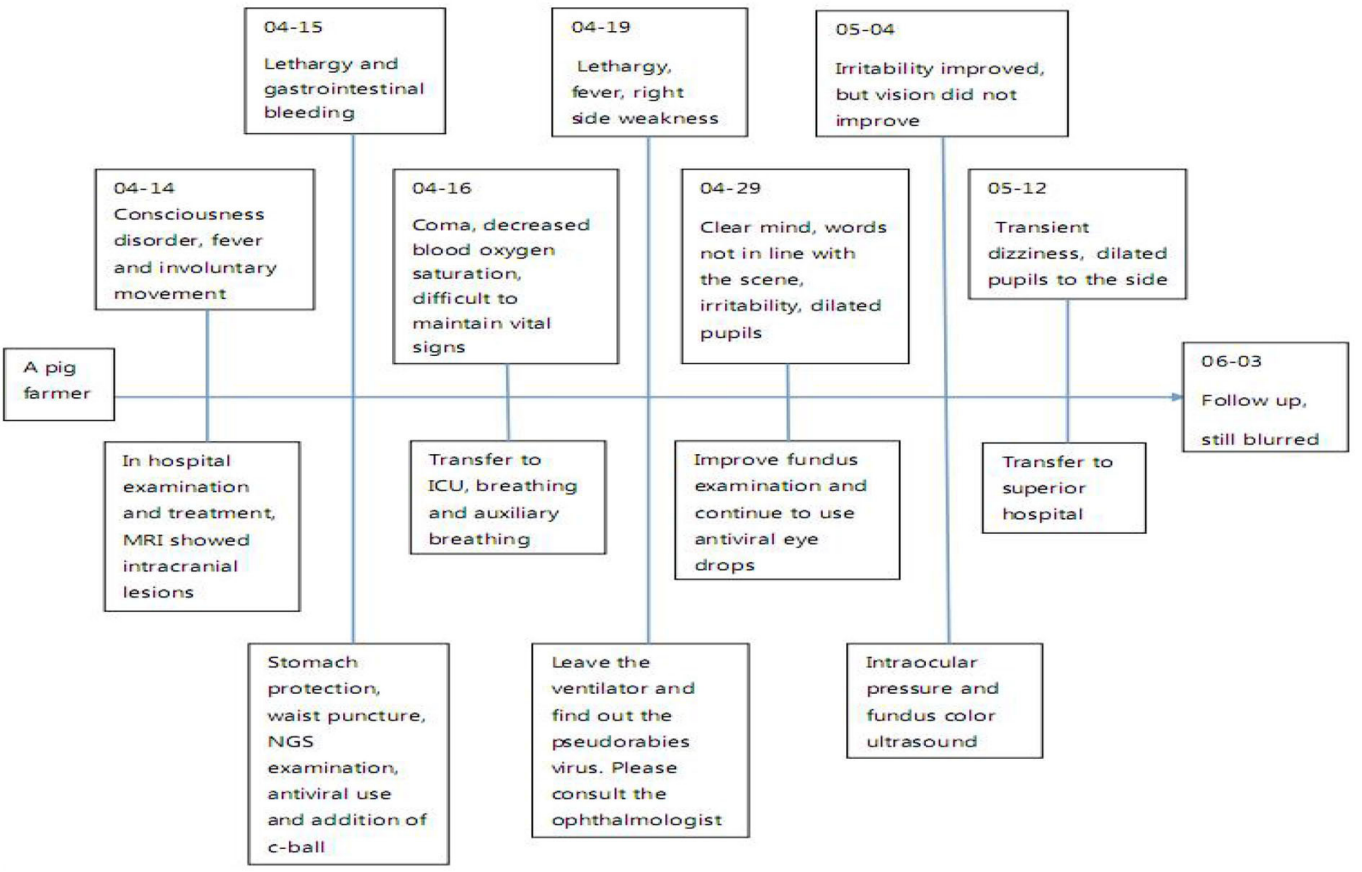
Timeline figure of the clinical information.

The current diagnostic criteria of viral encephalitis are as follows: 1) The change of mental state lasts for more than 24 h, except for other reasons; 2) Seizures, excluding previous history of epilepsy; 3) Body temperature > 38°C before or within 72 h after onset; 4) Emerging focal clinical manifestations of the nervous system; 5) CSF leukocyte > 5 × 10^6^/L; 6) Imaging suggests encephalitis; 7) Abnormal EEG indicates encephalitis. If more than 3 items are met, encephalitis can be considered in clinical diagnosis. The main features of this case: having a history of contact with pigs, acute onset, fever, epilepsy, disturbance of consciousness and visual impairment, requiring ICU for treatment, and even using a ventilator to assist breathing. (Figure 3) The cerebrospinal fluid examination was consistent with the characteristics of viral encephalitis. Brain magnetic resonance imaging showed T2WI high signal. NGS detected pseudorabies virus from cerebrospinal fluid. Although the PRV coverage was relatively low, the mapped readings were evenly distributed in the whole PRV genome. Based on the above characteristics, it was diagnosed as pseudorabies virus encephalitis, whose clinical manifestations should be differentiated from autoimmune encephalitis, optic neuromyelitis, Vogt-Koyanagi-Harada syndrome (VKH), lymphoma, and other diseases (10).

In terms of treatment, previous research reports suggest that to avoid possible delays, antiviral treatment should be given as soon as possible. Delaying antiviral treatment for more than 48 h may lead to poor prognosis, including ocular complications—acute retinal necrosis syndrome (11). Although after onset, the patient was given Ganciclovir antiviral and treated with Dexamethasone to resist inflammation and Gamma globulin to remove harmful antibodies at the first time, the condition was still aggravated and developed respiratory failure, and required ventilator to assist respiratory treatment. About half a month after the course of the disease, the patient had obvious visual impairment. Finally, although encephalitis was controlled, the visual impairment was difficult to reverse. In addition, the patient had obvious transient hyperbilirubinemia in the early stage of onset, it was not clear whether it was related to acute viral infection. Transient damage to liver function occurred in the course of the disease, which improved after liver protection treatment, and the mechanism needs to be studied.

## Conclusion

PRV is a newly discovered cross-species transmission virus. It has the characteristics of acute onset, rapid progression, severe nervous system damage, prone to respiratory failure, visual impairment, etc., and the disease has high morbidity and mortality. Early diagnosis and timely treatment can achieve certain curative effects. NGS is a rapid and accurate method to detect PRV infection. Therefore, in clinical work, if a patient with similar clinical manifestations to the patient in this article is admitted, it is necessary to ask in detail whether there is a history of feeding pigs and pork processing, and to carry out serological tests, especially pathogenic NGS tests, in order to exclude pseudorabies viral encephalitis. At the same time, it also reminds employees engaged in animal husbandry that they need to raise their awareness of self-protection.

## Data availability statement

The datasets presented in this article are not readily available because of ethical and privacy restrictions. Requests to access the datasets should be directed to the corresponding author.

## Ethics statement

Written informed consent was obtained from the patient for the publication of any potentially identifiable images or data included in this article.

## Author contributions

YB and HH drafted the manuscript, sorted out the clinical data of patients, consulted the literature, and reviewed English grammar. NW, HA, WH, and YB conducted a follow-up visit to the patients before and after treatment, collected detailed clinical data, and reviewed the manuscript. YB and JC reviewed and proofread the whole article. All authors contributed to the article and approved the submitted version.

## Conflict of interest

The authors declare that the research was conducted in the absence of any commercial or financial relationships that could be construed as a potential conflict of interest.

## Publisher's note

All claims expressed in this article are solely those of the authors and do not necessarily represent those of their affiliated organizations, or those of the publisher, the editors and the reviewers. Any product that may be evaluated in this article, or claim that may be made by its manufacturer, is not guaranteed or endorsed by the publisher.

## References

[1] Wang G Zha Z Huang P Sun H Huang Y He M. Structures of pseudorabies virus capsids. Nat Commun. (2022) 13:1–11. 10.1038/s41467-022-29250-3 35318331 PMC8940892

[2] Liu Q Wang X Xie C Ding S Yang H Guo S. A novel human acute encephalitis caused by pseudorabies virus variant strain. Clin Infect Dis. (2021) 73:e3690–700. 10.1093/cid/ciaa987 32667972

[3] Mravak S Bienzle U Feldmeier H Hampl H Habermehl KO. Pseudorabies in man. Lancet. (1987) 329:501–2. 10.1016/S0140-6736(87)92105-2 2881053

[4] Ai J W Weng S S Cheng Q Cui P. Human endophthalmitis caused by pseudorabies virus infection, China, 2017. Emerg Infect Dis. (2018) 24:1087. 10.3201/eid2406.171612 29774834 PMC6004832

[5] Zhao WL Wu YH Li HF Li SY Fan SY Wu HL. Clinical experience and next-generation sequencing analysis of encephalitis caused by pseudorabies virus. Zhonghua yi xue za zhi. (2018) 98:1152–7. 10.3760/cma.j.issn.0376-2491.2018.15.006 29690727

[6] Yang H N Han H Wang H Cui Y Liu H Ding S. A case of human viral encephalitis caused by pseudorabies virus infection in China. Front Neurol. (2019) 10:534. 10.3389/fneur.2019.00534 31214104 PMC6558170

[7] Wang D Tao X Fei M Chen J Guo W Li P. Human encephalitis caused by pseudorabies virus infection: a case report. J Neurovirol. (2020) 26:442–8. 10.1007/s13365-019-00822-2 31898060 PMC7223082

[8] Fan S Yuan H Liu L Li H Wang S Zhao W. Pseudorabies virus encephalitis in humans: a case series study. J Neurovirol. (2020) 26:556–64. 10.1007/s13365-020-00855-y 32572833

[9] Zhou Y Nie C Wen H Long Y Zhou M Xie Z. Human viral encephalitis associated with suid herpesvirus 1. Neurol Sci. (2022) 43:2681–92 10.1007/s10072-021-05633-0 PMC8514202 34647219

[10] Xiao J Gao L Zhang M Wang X Xuan J Liu G. Clinical features of diffuse leptomeningeal glioneuronal tumor with rapid blindness misdiagnosed as NMOSD and literature review. SN Compr Clin Med. (2019) 1:434–41. 10.1007/s42399-019-00058-5

[11] Yan W Hu Z Zhang Y Wu X Zhang H. Case report: metagenomic next-generation sequencing for diagnosis of human encephalitis and endophthalmitis caused by pseudorabies virus. Front Med. (2021) 8:753988. 10.3389/fmed.2021.753988 35096860 PMC8795075

